# From Deep Mutational Mapping of Allosteric Protein Landscapes to Deep Learning of Allostery and Hidden Allosteric Sites: Zooming in on “Allosteric Intersection” of Biochemical and Big Data Approaches

**DOI:** 10.3390/ijms24097747

**Published:** 2023-04-24

**Authors:** Gennady Verkhivker, Mohammed Alshahrani, Grace Gupta, Sian Xiao, Peng Tao

**Affiliations:** 1 Keck Center for Science and Engineering, Graduate Program in Computational and Data Sciences, Schmid College of Science and Technology, Chapman University, Orange, CA 92866, USA; alshahrani@chapman.edu (M.A.); grgupta@chapman.edu (G.G.); 2 Department of Biomedical and Pharmaceutical Sciences, Chapman University School of Pharmacy, Irvine, CA 92618, USA; 3 Department of Chemistry, Center for Research Computing, Center for Drug Discovery, Design, and Delivery (CD4), Southern Methodist University, Dallas, TX 75275, USA; sxiao@smu.edu (S.X.); ptao@smu.edu (P.T.)

**Keywords:** allosteric regulation, deep mutational scanning, molecular dynamics, protein stability, network modeling, deep machine learning, binding energetics, allosteric communications, allosteric binding pockets

## Abstract

The recent advances in artificial intelligence (AI) and machine learning have driven the design of new expert systems and automated workflows that are able to model complex chemical and biological phenomena. In recent years, machine learning approaches have been developed and actively deployed to facilitate computational and experimental studies of protein dynamics and allosteric mechanisms. In this review, we discuss in detail new developments along two major directions of allosteric research through the lens of data-intensive biochemical approaches and AI-based computational methods. Despite considerable progress in applications of AI methods for protein structure and dynamics studies, the intersection between allosteric regulation, the emerging structural biology technologies and AI approaches remains largely unexplored, calling for the development of AI-augmented integrative structural biology. In this review, we focus on the latest remarkable progress in deep high-throughput mining and comprehensive mapping of allosteric protein landscapes and allosteric regulatory mechanisms as well as on the new developments in AI methods for prediction and characterization of allosteric binding sites on the proteome level. We also discuss new AI-augmented structural biology approaches that expand our knowledge of the universe of protein dynamics and allostery. We conclude with an outlook and highlight the importance of developing an open science infrastructure for machine learning studies of allosteric regulation and validation of computational approaches using integrative studies of allosteric mechanisms. The development of community-accessible tools that uniquely leverage the existing experimental and simulation knowledgebase to enable interrogation of the allosteric functions can provide a much-needed boost to further innovation and integration of experimental and computational technologies empowered by booming AI field.

## 1. Introduction

Over the past years, artificial intelligence (AI) and machine learning (ML) approaches have increasingly found their way into biomedical research, healthcare applications, and drug discovery. AI augments the capabilities of human researchers, often outperforming alternative methods, and leverages large amounts of high-quality data obtained in biochemical and biophysical experiments for model training which is a key bottleneck that can unlock the full potential of AI technologies. The advances in the ML field have driven the design of new tools and systems to model increasingly complex chemical and biological phenomena [1,2,3,4,5]. Recent years have witnessed the remarkable growth of data-intensive experimental high-throughput technologies and machine learning tools aiming for atomistic-level interrogation and characterization and engineering of protein functions and mechanisms. Allosteric interactions and communications in proteins are central to diverse biological mechanisms, and allosteric regulation phenomenon has been long recognized as the ‘second secret of life’ [6,7,8,9,10,11,12,13,14]. Nonetheless, dynamic molecular mechanisms that give rise to allosteric molecular events continue to be fairly elusive and are often characterized at a phenomenological level, lacking rigorous and universal theoretical and computational foundation that can explain the enormous diversity of these processes. Diverse spatial and temporal scales of thermodynamic and dynamic changes underlie allosteric regulatory mechanisms and communications. Among important reasons that limited the fundamental progress in characterization of allosteric mechanisms across human proteome is the lack of precise and robust high-throughput biochemical methods that can comprehensively quantify allosteric effects at the residue level resolution and the sparse data on quantitative mappings of the genotype-phenotype landscapes for allosterically regulated proteins.

**Figure 1:**
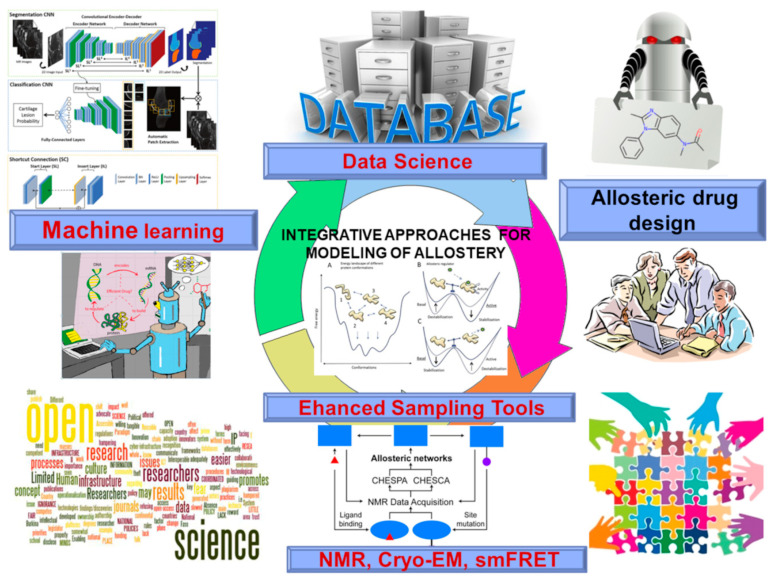
The integrative computational and experimental approaches for studies of conformational dynamics and allosteric regulation mechanisms. (A) The energy landscape of different allosteric conformations. (B,C) Different effects of allosteric regulators on modulating the thermodynamic equilibrium between allosteric conformations.

Structural and biophysical studies of allosteric molecular events that involve the formation of short-lived allosteric states and intermediates have benefited from advances in nuclear magnetic resonance (NMR) technologies that can be employed as powerful tools for probing of allosteric interactions and communications in proteins [15,16,17,18]. NMR studies highlighted progress in the determination of hidden allosteric states [19,20,21,22,23,24,25,26]. AI and ML approaches can capitalize on the growing experimental and simulation data on protein dynamics to enable robust exploration of conformational landscapes for complex biomolecular systems [27,28,29,30,31,32,33,34,35]. In our recent review, we discussed potentials and limitations of integrative computational and experimental studies empowered by AI/ML tools for quantitative characterization of allostery from first principles [36] focusing on applications of ML approaches for efficient conformational sampling and characterization of protein dynamics. In the present review, we examine and discuss in detail new developments along two major directions of allosteric research through the lens of data-intensive biochemical approaches and AI-based computational methods (Figure 1). We focus on the latest remarkable progress in deep high-throughput mining and comprehensive mapping of allosteric protein landscapes and allosteric regulatory mechanisms. This review also analyzes the latest developments in AI and ML methods for atomistic modeling of allosteric proteins, experiment-guided mapping of allosteric energy landscapes, and new predictive models of allosteric binding sites on the proteome level (Figure 1). We also discuss the recent progress in the AI-augmented structural biology approaches that empower and expand high-throughput capabilities of X-ray crystallography, cryo-electron microscopy (cryo-EM), and single-molecule Förster resonance energy transfer (smFRET) technologies to expand our knowledge of the universe of protein dynamics and allostery. We conclude with an outlook and highlight the importance of developing an open science infrastructure for machine learning studies of allosteric regulation and validation of computational approaches using integrative studies of allosteric mechanisms.

## 2. Mining of Allosteric Protein Landscapes and Mechanisms Using Multidimensional Deep Mutational Scanning Approaches

**Figure 2:**
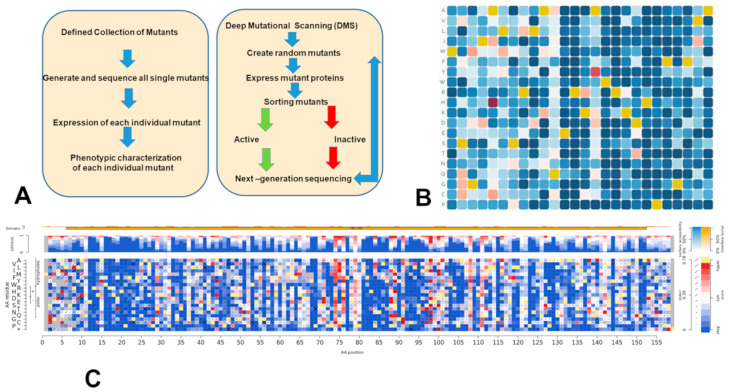
(**A**) Mutational profiling approach is typically characterized by a defined collection of single mutants. In the DMS approach, the generation and expression of random mutants are followed by high-throughput sorting of active mutants from inactive mutants using flow cytometry, phage display, or growth selection. (**B**) An example of visualization of deep mutational scanning data from MaveDB (https://www.mavedb.org/, accessed on 10 April 2023). MaveDB *is* an open public repository for datasets from Multiplexed Assays of Variant Effect (MAVEs), such as those generated by DMS [46]. Each cell in the heatmap is a different genetic variant measured in the experiment, where the color corresponds to the functional impact of the variant. (**C**) An example of DMS visualization for the human SUMO E2 conjugase UBE2I using functional complementation in yeast via DMS-TileSeq obtained from *MaveDB* [46]. DMS-TileSeq read counts were used to establish relative allele frequencies in each condition. Non-mutagenized control counts were subtracted from counts (as estimates of sequencing error). log ratios of selection over non-selection counts were calculated. The resulting TileSeq fitness values were normalized to a 0–1 scale, where 0 corresponds to the median nonsense variant score and 1 corresponds to the median synonymous score [47].

Recent biochemical studies exploited and further advanced the Deep Mutational Scanning (DMS) approach to enable direct mapping of residue-based biophysical effects of mutations on protein stability, binding, and expression (Figure 2) [37,38,39,40,41,42,43,44,45,46,47,48,49]. Biochemical and functional studies using protein engineering and deep mutagenesis have quantified binding mechanisms of SARS-CoV-2 interactions with the host receptor ACE2 and a wide range of antibodies [38,39,40,41,42,43]. The landscapes of antibody binding for SARS-CoV-2 spike protein variants were systematically mapped by measuring the binding affinity of all possible combinations of these 15 receptor binding domains (RBD) mutations to a panel of monoclonal antibodies with distinct epitopes [42]. A powerful DMS platform based on non-replicative pseudotyped lentiviruses quantified how SARS-CoV-2 spike mutations impact pseudo-virus infection by providing a high-throughput approach to measure the effect of ∼10^5^ combinations of mutations on antibody neutralization [43]. 

A pioneering approach for global mapping of the energetic and allosteric landscapes of protein interaction domains leverages the massively parallel nature of DMS and employs multidimensional mutagenesis to causally quantify the phenotypic effects of thousands of perturbations for multiple molecular phenotypes in diverse genetic backgrounds [44]. The new technique known as Double Deep Protein-Fragment Complementation Assay (ddPCA) employs two separate selection assays based on the protein-fragment complementation (PCA) assay so that one assay can detect mutational effects on the stability of the protein (AbundancePCA) while the other assay detects both stability and protein–protein interactions (BindingPCA). Compared to other experimental approaches, ddPCA can disentangle the stability and binding affinity effects of mutations and systematically quantify allosteric sites that are distant from the binding interface in which mutations induce significant changes on the binding via long-range [44]. These illuminating studies have provided invaluable insights into the underlying global features of allosteric landscapes that are driven by evolutionary requirements for multiple phenotypic fitness factors. Among common “rules” of allostery emerged from this study was the realization that allosteric mutations and allosteric sites are common in protein domains and the frequency of allosterically influential positions can increase near the protein binding interfaces, suggesting that local allosteric perturbations may present an important mechanism of allosteric modulation of binding [44]. Remarkably, this study also discovered the abundance of mutations in both the protein core and protein surfaces that can alter stability and binding affinity, which signified presence of a large genotypic space available for evolution and modulation of diverse allosteric regulatory mechanisms.

In a very recent follow-up study, Lehner and coworkers developed a comprehensive map of allostery and reported multiple global atlases of the inhibitory allosteric communications in KRAS protein by measuring the impact of mutations on the protein folding and binding to six interaction partners [45]. By using the developed enhanced DMS technologies, this study systematically quantified and disentangled mutational effects of protein residues in different genetic backgrounds which allowed to infer biophysical effects of >26,500 variants of KRAS. In addition, this approach reconstructed the free energy landscapes of KRAS binding with six interaction partners by producing >22,000 precise thermodynamic measurements of the mutation-induced free energy changes—a truly monumental achievement and a revolutionary step towards a comprehensive atomistic mapping of genotype-phenotype landscapes on a large scale [45]. In particular, this study quantified the inhibitory allosteric landscape for RAF1 binding by discovering a total of 361 allosteric mutations in 74 distally located from the binding interface allosteric positions, showing that allosteric mutations are highly enriched in the nucleotide binding pocket site of KRAS. This remarkable study revealed principles of allosteric communication in KRAS which may be generalizable to other signaling proteins, showing that allosteric mutations can inhibit binding to all three KRAS effectors and allosterically control binding specificity indicative of fundamental role of allosteric sites in modulation and fine-tuning of binding, regulation, and signaling [45].

DMS studies of the FabZ, LpxC, and MurA proteins that are involved in cell envelope synthesis were performed with the aid of high-throughput CRISPR genome editing, revealing the effect of >17,000 protein variants on function [48]. Mutational libraries generated in this study were deployed to characterize resistance development against antimicrobial compounds that target the selected protein, dissecting the role of specific amino acids in supporting viability. Nonetheless, this powerful approach displayed certain limitations by assessing the effects of single mutations while synergistic and compensatory effects of multiple mutations arising during evolution or due to protein responses eliciting drug resistance remained elusive [48]. The importance of nonadditive, epistatic relationships was assessed in the systematic biochemical analysis of all mutants at the binding interfaces of the SARS-CoV-2 spike variants [49]. A systematic DMS-based mapping of the epistatic interactions between the SARS-CoV-2 Spike BA.1 variant mutations relative to the Wu-Hu-1 original strain showed evidence of compensatory epistasis in which immune escape mutations are compensated by the emergence of affinity-enhancing mutations with the host cell receptor ACE2 [50]. While mutations in proteins are typically deleterious and can impair to a different degree protein function, and stability, compensatory neutral mutations often facilitate protein evolution through positive epistasis with deleterious mutations in the same protein. By enabling high-throughput quantitative analysis of functional effects for thousands of mutations, DMS tools can facilitate understanding of complex synergistic mechanisms and dissect functional cross-talks between mutational sites in addition to quantifying the impact of single mutations. This venue presents a new frontier for further development of DMS technologies, a challenge that requires integration of the DMS datasets [47] with emerging ML models of epistasis and allostery [51].

A high-throughput platform was developed for DMS analysis of caspase-3 (CASP3) and caspase-7 (CASP7) allowing for a large-scale assessment of mutations on caspase structure and function and revealing differences between CASP3 and CASP7 proteolytic activity and protein stability [52]. This study highlighted important limitations of “generic” DMS studies in differentiating between specific molecular mechanisms as mutations that decrease caspase activity could impact functional changes across multiple phenotypes, including protein expression, stability, catalytic activity, substrate specificity, and allosteric regulation. Strikingly, the results of this study further underscored the unique nature of Lehner’s studies [45] that brilliantly overcame these inherent biophysical uncertainties in their ddPCA-based DMS assays and systematically quantified specific functional consequences of every mutation in the system resolving biophysical ambiguities that are inherently present in standard DMS experiments.

Allosteric mapping of GTPase switch Gsp1/Ran system in the context of its in vivo protein interactions by comprehensive mutational scanning enabled characterization of allosteric sites for highly regulated molecular switches that catalyze GTP hydrolysis and nucleotide exchange [53]. Using a DMS experimental platform, this study discovered new regulatory sites that are allosterically coupled with the GTPase active site allowing allosteric mutations to alter Gsp1/Ran cellular function through modulation of GTPase switching. A large-scale mapping of the genotype-phenotype landscape for the lac repressor, LacI quantified the effect of mutations on LacI allostery, showing that a complex balance between contributions from many residues and interactions can ultimately drive the phenotypic outcomes [54]. Consistent with other studies, it was found that allosteric genotype-phenotype landscapes can allow for rapid evolutionary innovation resulting in novel allosteric phenotypes emerging from sampling a broader genotype space with higher-order mutations [54]. The range and experimental details of DMS methods with a specific focus on recently developed techniques in mutation library generation, high-throughput methods, and data analysis were discussed in a recent insightful review [55].

## 3. Machine Learning Models Infer Genotype-Phenotype Relationships from DMS Experiments

ML models leveraged the data-rich DMS results to generate predictive models and obtain mechanistic insights into genotype-phenotype maps [56,57,58,59,60,61]. A positive-unlabeled (PU) learning framework can infer sequence-function relationships from large-scale DMS data showing strong predictive performance across ten large-scale sequence-function datasets, representing proteins of different folds, functions, and library types [56]. DMS experiments were combined with molecular dynamics (MD) simulations and network analysis to examine the functional landscape of a bacterial transcription factor showing the role of diverse and broad ensembles of mutational communication pathways in propagating allosteric phenotypic effects [57]. Deep learning (DL) models built upon DMS data for several bacterial allosteric transcription factors predicted the distribution of allosteric hotspots revealing that regulatory sites mediating allostery can be broadly distributed on the protein rather than being confined to narrow pathways linking the allosteric and active sites [58]. An important revelation of this study was the ability of DL models to accurately predict functional sites and link these predictions with molecular mechanisms of allosteric regulation. A supervised DL architecture helped to infer sequence–function relationships using training on large-scale DMS datasets [59]. Using different network architectures ranging from linear regression to convolutional networks, this study displayed a predictive accuracy on five diverse DMS datasets and enabled the design of a protein sequence that binds to immunoglobulin G with substantially enhanced affinity compared to the wild-type GB1 [59]. A statistical learning framework was developed to infer the protein fitness landscape for DHFR protein from directed evolution data in which the inferred landscape model revealed numerous examples of epistasis arising from interactions between residues and allow for analysis of evolutionary trajectories for protein engineering [60]. Recent studies showed that ML models can successfully reconstruct sequence–function landscapes from large datasets and facilitate efficient protein engineering with less experimental screening than traditional directed evolution approaches [61]. Understanding the relationship between promoter sequence, expression phenotype, and fitness are central in understanding evolution and gene regulation. The genomic analysis of sequence-to-expression regulatory fitness landscapes leveraged data on millions of DNA sequences and their experimentally measured expression to develop ML models and transformer encoders that represented each sequence using a sorted vector of expression changes, allowing for large scale exploration of novel genotypes and predictions of expression levels [62].

Multiplex assays of variant effect (MAVEs) are a type of DMS experiment that utilize a combination of reporter assays, protein expression assays, and next-generation sequencing (NGS) to screen thousands of variants in a single experiment and can be used to study the effect of genetic variants on gene expression, protein stability, and protein-protein interactions. Quantitative modeling of MAVE data is based on the use of latent phenotype models, which describe the relationship between the observed data and a latent phenotype. The framework is designed to capture the complex structure of MAVE data, including both the observed phenotype data and the underlying latent phenotype. The latent phenotype model is then used to estimate the latent phenotype from the observed data, and to quantify the effect of different environmental factors on the underlying latent phenotype [63]. Using this framework, a Python package MAVE-NN implements a range of ML models for learning genotype-phenotype maps using latent phenotype neural networks and parameters inferred from MAVE data using a TensorFlow 2 backend [63]. With the improving accuracy of DMS datasets and the enhanced predictive power and robustness of generative ML models, the integrative tools combining these approaches can discover functional protein sequences with the designed fitness profile and guide exploration towards effective protein engineering solutions.

## 4. Learning Protein Conformational Dynamics Using AI Approaches

Exploring and learning the protein allostery universe becomes increasingly plausible using the proliferation of AI/ML tools and methods to solve long-standing problems in molecular biology and biophysics. The remarkable success of AlphaFold2 (AF2) in protein structure modeling and looming resolution of the protein folding problem in the last few years have marked a revolutionary change in structural biology that was inconceivable even several years ago [64,65]. However, the key shortcomings of the AF2 technology in resolving conformational dynamics and allosteric phenomenon challenges are real and are mainly associated with the limitations to accurately capture the ensembles and energetics of functional conformational changes as well as mutational effects on protein dynamics and functions [66]. In recent illuminating reviews, Nussinov and colleagues noted that expanding AF2 tools for prediction of allosteric regulatory mechanisms would require a considerable step forward beyond an accurate single structure prediction—from mapping of the conformational ensembles to detection of low-populated allosteric conformations and hidden functional states [67,68]. Several recent studies have provided strong evidence that structure prediction capabilities of the AF2 methods are not trivially expandable for the prediction of complex allosteric landscapes and mechanisms of allosteric regulation [69,70]. These formidable challenges will test the limits and further accelerate the developments of AI technologies that are currently incorporating new concepts and architectures—from natural language processing to generative transfer learning to enable unprecedented in its scale prediction and design of protein structure, functions, and mechanisms. The latest breakthrough in AI-based applications to large-scale protein structure predictions involved language models of protein sequences at the scale of evolution trained on up to 15 billion parameters that resulted in high accuracy end-to-end family of transformer protein language models, ESMFold for atomic level structure prediction directly from the individual sequence of a protein [71]. Compared to AF2, the trained language model directly learns at scale evolutionary patterns of sequences linked to structure, strikingly eliminating the need for the evolutionary databases, multiple sequence alignments, and templates. This results in a speed improvement for structure prediction of more than an order of magnitude while maintaining high resolution accuracy comparable to AF2 models [71]. A new method for accurate protein design by integrating structure prediction networks and diffusion generative model called RoseTTAFold Diffusion (RFdiffusion) enables the design of complex functional proteins from simple molecular specifications [72]. This method can generate novel protein structures of more than 600 residues that are accurately predicted by AF2 and ESMFold, with the experimental data demonstrating that designs are extremely thermostable [72]. These truly revolutionary developments in the development and application of innovative AI technologies to fundamental challenges in protein science and molecular biology are driven by unified efforts from a myriad of academic laboratories and are accelerated by the growing attention to these problems and competition between industry giants DeepMind/Google and Meta. 

The biological Importance and complexity of protein conformational dynamics and allosteric mechanisms require next-generation computational approaches and innovative AI-based integrative tools synergizing advances in computation and experiments that can enable atomistic characterization of allosteric landscapes, states, and interactions. Development of AI-augmented simulation approaches that leverage ML tools to represent physics-based thermodynamic and kinetic determinants of conformational sampling as deep neural networks has gained a significant momentum in recent years. ML approaches were employed to facilitate exploration of conformational landscapes using MD simulations via optimal selection of reaction coordinates. By combining deep learning (DL) approaches and biased MD simulations, physically meaningful collective variables can be determined to resolve the bottlenecks that often hinder the reliable characterization of conformational transitions and rare events [73]. Utilizing variational inference and a predictive information bottleneck concept implemented through deep neural networks, a new framework was recently proposed that utilizes brief MD simulations to estimate the reaction coordinates and to conduct iterative biased simulations, which enables improved exploration of conformational landscapes and reliable prediction of the corresponding thermodynamic and kinetic features of the system [74,75,76,77]. The ML model can be trained with simulation data to generate realistic conformational ensembles of proteins without sampling. A recently introduced generative adversarial network, idpGAN, based on a transformer architecture with self-attention on coarse-grained simulations can be used to train an ML model to generate physically realistic conformational ensembles of proteins without needing to sample data and with minimal computational cost [78]. This proof-of-principle model can predict sequence-dependent conformational ensembles for sequences that are not present in the training set and can be further generalized for atomistic ensembles.

**Figure 3:**
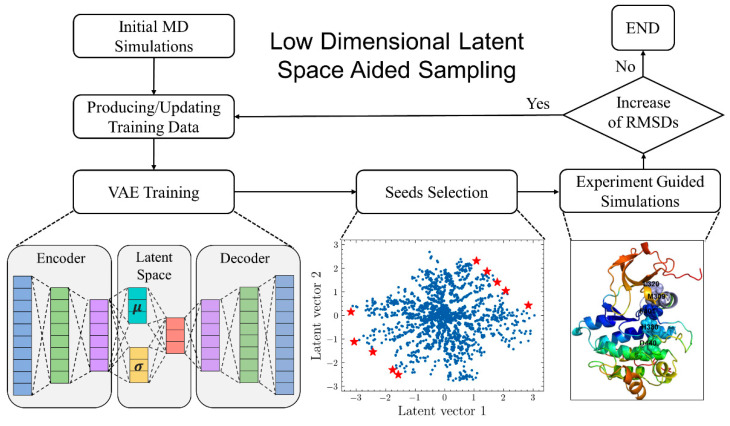
The architecture of latent space-assisted adaptive sampling method for protein trajectories LAST [84] includes cycles of VAE training, seeding structure selection on the latent space, and conformational sampling through iterative cycles of short MD simulations. The proposed approach is validated using simulation studies of metastable states of adenosine kinase (ADK) and native states of Vivid protein (VVD). The red stars refer to different protein conformations in the latent space of the model.

VAMPNets, an end-to-end learning system, can be used to predict molecular kinetics with neural networks [79]. The combination of VAMPNet and graph-level dynamics with neural networks creates GraphVAMPNet, which can accurately identify high resolution metastable states from long-term MD trajectories [80]. GMVAE can be used to construct a reduced representation of the free energy landscape of protein folding, which has clusters that correspond to the metastable states during folding [81]. A method that applies ML models and MD simulations to explore protein conformational space uses an autoencoder to map MD snapshots onto a user-defined conformational landscape defined by principal components analysis or specific structural features [82]. Variational auto encoders (VAE) models were adapted for encoding protein structures into a two-dimensional latent space to describe conformational changes between different allosteric states [83]. The recently introduced latent space-assisted adaptive sampling method for protein trajectories (LAST) [84] can accelerate the exploration of protein conformational space by using VAE and exploitation of the iteratively optimized training data for the latent-based sampling (Figure 3). Using enhanced simulation schemes and machine learning models, researchers have studied the molecular factors that lead to allosteric changes and ligand-induced ensemble changes in proteins. Linear Discriminant Analysis was used to identify differences between the apo and allosteric inhibitor-bound ensembles [85], and another ML was developed to understand the temporal relationships of allosteric stimulation in hemagglutinin-neuraminidase [86]. Zhou and colleagues employed an allosteric community model based on a machine learning method to examine the allosteric mechanism of the Vivid (VVD) protein [87]. An autoencoder-based detection method for characterizing ligand-induced dynamic allostery was used to compare the time fluctuations of protein structures in the form of distance matrices acquired from MD simulations [88].

## 5. From AI-Enabled Mapping of Allosteric Conformational Landscapes to ML Discovery of Cryptic Allosteric Sites

The nature and atomistic details of the allosteric communication between the allosteric site and the functional site are often difficult to dissect. While experimental approaches could reveal allosteric hotspots and potential communication pathways in protein structures, robust and large scale-based tools for mapping of allosteric communications and allosteric binding sites are still missing. The problem of identifying putative allosteric sites can be addressed by considering the communication with orthosteric sites and uncovering the allosteric communication pathways between orthosteric and allosteric sites. Computational methods for predicting binding sites and allosteric pockets can be classified as geometric, energetic, evolution-based, knowledge-based, and machine learning approaches. AlloPred uses normal mode perturbations along with pocket descriptors in a machine learning approach to rank allosteric sites, with a good performance of 28 out of 40 cases [89]. The Allosite approach, which uses support vector machine classifiers of topological and physicochemical characteristics of allosteric and non-allosteric sites, has shown promise in predicting allosteric pockets [90]. AllositePro, an extension of Allosite, combines pocket features with perturbation analysis to identify allosteric sites and was able to validate a novel allosteric site in cyclin-dependent kinase 2 (CDK2) [91]. The Allosite and AllositePro tools, along with the Alloscore scoring function and Allosterome database service, have been essential components of allosteric services and applications developed in the past decade for the evaluation of allosteric protein–modulator interactions and evolutionary analysis of query allosteric sites/modulators within the human proteome [92]. The Active and Regulatory site Prediction (AR-Pred) model was developed by utilizing various residue-based features such as amino acid physicochemical properties, rate of residue evolution, and features describing protein geometry and dynamics [93].

Recently, autonomous predictors of binding sites based solely on ML methods have emerged, replacing traditional template-free methods such as Fpocket [94], SiteHound [95], and MetaPocket 2.0 [96]. ISMBLab-LIG is an ML algorithm that uses 3D probability density maps of interacting atoms as input attributes to predict ligand binding sites (LBSs), which are relatively insensitive to local conformational variations [97]. DeepSite utilizes 3D voxelization of the protein and deep convolutional neural networks (DCNN) for binding site prediction, outperforming other structure-based algorithms on a set of 7622 proteins from the scPDB database [98]. Other ML-based approaches such as FRSite [99] and Kalasanty [100] also use 3D voxelization of the entire protein, with FRSite based on Faster-RCNN and Kalasanty employing a common segmentation architecture U-Net.

In recent years, the concept of end-to-end learning has been gaining attention. This approach involves three main steps: (a) reducing the dimensions of inputs, (b) creating various neural networks based on the available data, and (c) applying backpropagation across the entire architecture to reduce loss and update weights. For example, a neural relational inference model that utilized a graph neural network and an autoencoder architecture was used to explore the latent embedding of allosteric systems, learn about the long-range interactions and communication between distant sites in the ligand-induced allosteric regulation of Pin1, conformational transition of SOD1 protein, and the activation of MEK1 caused by oncogenic mutations [101]. Another machine learning (ML) approach, referred to as DiffNets, was proposed which does not assume whether large structural changes are more important than local changes, rather it utilizes a self-supervised autoencoder to learn features of the conformational ensembles which are pertinent to distinguish between protein systems [102]. DeepSurf introduces a new representation of the 3D protein surface based on local voxel grids located around the surface, as well as a novel residual network, LDS-ResNet, which has been extended in three dimensions to be applicable to volumetric data [103]. This proposed method has been assessed in terms of binding site prediction using various benchmark datasets, evidencing its superiority when compared to other state-of-the-art approaches.

A template-free P2Rank approach is among the most efficient and fast available ML tools for prediction of ligand binding sites [104,105]. It combines information from both sequence and structural data to rank potential binding sites based on their likelihood of binding a specific ligand. P2Rank uses support vector machine (SVM), random forests (RF), and artificial neural networks (ANNs) to learn the features that are most important for binding site prediction. This method is based on the prediction of the ligandability of a local chemical environment that is centered on points placed on the protein’s solvent-accessible surface [104,105]. P2Rank works by scoring and clustering these points on the protein’s surface. The ligandability score of individual points is determined by a machine learning model that is trained on a dataset of known protein–ligand complexes. The P2Rank approach assigns structural, physical-chemical, and evolutionary features to points on a mesh that covers the protein surface and builds a ML model from this representation. Rather than learning to memorize ligand binding sites, P2Rank learns what makes local neighborhoods around the protein surface intrinsically ligandable. This then allows the detection of ligandable points, which are then clustered to produce a list of surface patches corresponding to the predicted binding sites [104,105].

Recent advances in convolutional neural networks (CNNs) have made them very effective in image recognition tasks. They have been widely applied to detect protein binding sites which are represented as 3D-voxels with atomic attributes. A novel, rapid, and accurate DL approach, BiteNet (Binding site neural Network) was developed for large-scale and spatiotemporal identification of protein binding sites. Drawing inspiration from computer vision problems such as object detection in images and video, BiteNet considers protein conformations as three-dimensional images with channels corresponding to the atomic densities, binding sites as the objects to detect on these images, and conformational ensembles of proteins as three-dimensional videos to analyze [106]. In comparison to the more established techniques, BiteNet has the ability to enable an extensive examination of conformational ensembles and has the potential to recognize allosteric binding sites in both soluble and transmembrane protein domains. The method has demonstrated its efficacy in identifying the most challenging binding site detection problems on three-dimensional structures of pharmacological targets, such as ATP-gated cation channels, epidermal growth factor receptors (EGFRs), and G protein-coupled receptors (GPCRs). Notably, it successfully identified an oligomer-specific allosteric binding site made up of the subunits of the trimeric P2X3 receptor complex, as well as a conformation-specific allosteric binding site of the epidermal growth factor receptor kinase domain [106]. This method was validated on a large HOLO4K benchmark dataset of holo protein structures used for evaluation of binding site prediction methods [107] showing high accuracy and speed, outperforming both DeepSite and P2Rank tools. BiteNet offers an alternative to traditional methods of predicting binding sites in holo protein structures, by utilizing large-scale analysis of conformational ensembles to assess protein dynamics and flexibility. The sites of interest which are detected by this method can then be utilized for structure-based drug design.

**Figure 4:**
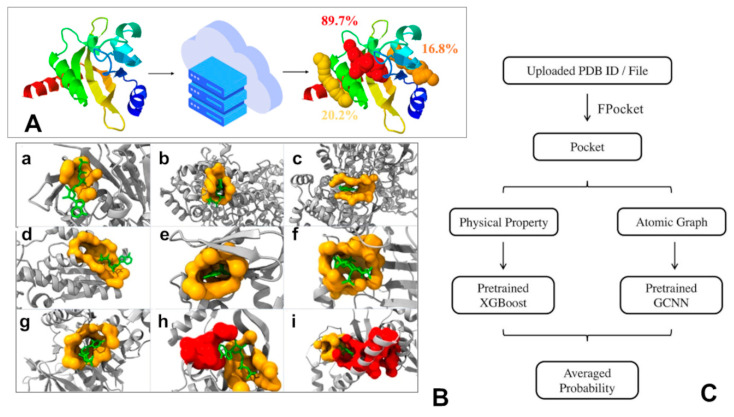
(**A**) A web server workflow for the Prediction of Allosteric Sites Server (PASSer) approach [108,109] for prediction of allosteric binding sites is presented. Users may submit a PDB ID or a PDB file, and the server will use a pretrained XGBoost model to calculate and predict physical properties for each pocket, and a pretrained GCNN model to construct and process an atomic graph. (**B**) Structures of nine proteins with modulators and predicted pockets [109]. The following protein structures were analyzed: (**a**) 2FPL, (**b**) 2R1R, (**c**) 3BCR, (**d**) 4PFK, (**e**) 1Q5O, (**f**) 3PEE, (**g**) 4HO6, (**h**) 1XMV, and (**i**) 2OZ6 [109]. The yellow pockets are labeled as allosteric, and the lime molecules are modulators. For (**a**–**g**), the allosteric pockets are successfully predicted as top one by our model. For (**h**,**i**), the red pockets are predicted as the first place, and the allosteric pockets are predicted as the second place. (**C**) A schematic flowchart of PASSer algorithm [108,109].

An ensemble learning method combining eXtreme gradient boosting (XGBoost) and graph convolutional neural networks (GCNNs), and an automated machine learning method (AutoGluon and AutoKeras) was developed to predict plausible allosteric sites and both models were incorporated to Prediction of Allosteric Sites Server (PASSer) (Figure 4) [108,109]. Most ML models are designed to make predictions either as labels or probabilities for all pockets detected in studied proteins, which is still a challenging and time-consuming task. Learning to Rank (LTR), an emerging area, and LTR models provide ‘relative’ predictions by ranking objects from the most to the least relevant, making detection and ranking of the allosteric sites more tractable. LambdaMART model is implemented for allosteric sites ranking with gradient boosting decision tree (GBDT) and Lambdarank loss function [110]. Compared with other ML models such as XGBoost, SVM, and RF, LambdaMART achieved the highest efficiency and showed a better ability to rank the experimentally known allosteric sites at top positions [110].

Cryptic binding sites are small and hidden pockets in proteins that are difficult to detect using traditional methods such as X-ray crystallography and NMR spectroscopy as they may emerge in meta-stable intermediate states that are typically short-lived and dynamic in nature, often appearing during allosteric conformational changes. Additionally, cryptic pockets may contain non-polar or hydrophobic residues that do not form strong hydrogen bonds with other molecules in the protein, making them difficult to detect by computational methods. Accurate and rapid discovery of potential cryptic pockets in a protein structure is an important and challenging problem and robust approaches capable of uncovering hidden druggable sites that can greatly accelerate the scope of therapeutic interventions and modernize drug discovery of the future. 

While the repertoire of available methods for detection of protein binding pockets and ligand binding sites is rich and diverse, the reliable identification of allosteric binding sites and hidden allosteric pockets available in short-lived intermediate states is far more challenging. The emergence of recent AI and ML models to incorporate the elements of protein dynamics and sophisticated CNN and DL architectures may provide a fruitful avenue for future developments. These directions are organically linked with the increasing interest to adaptation of AF2 tools for prediction of conformational ensembles and functional states with previously unknown hidden pockets making them an appealing target for a new generation of drug discovery technologies. An important step forward to address challenges of detecting cryptic binding pockets emerging from conformational ensembles is made in a novel method PocketMiner that employs DL-based architecture to identify pockets in large protein structures that have not been identified through crystallography or other experimental techniques. In contrast to previous methods, PocketMiner utilizes molecular simulations to evaluate if each residue in a protein structure has the ability to rearrange its orientation to participate in a cryptic pocket as part of its thermal fluctuations. The proposed DL model is trained on extensive datasets of structural ensembles derived from simulations that contain examples of pocket opening events. PocketMiner can detect hidden binding sites more than 1000 times faster than existing methods when applied to single structures from a newly created dataset of 39 experimentally confirmed cryptic pockets [111,112]. PocketMiner was also applied to the human proteome discovering that more than half of proteins, which were assumed to lack pockets based on the available structures, likely contain cryptic pockets [111,112].

## 6. AI-Augmented Cryo-EM and smFRET Approaches Expand the Universe of Protein Conformational Dynamics

Recent advances in single-particle cryo-electron microscopy (cryo-EM) have enabled the determination of numerous near-atomic resolution structures for well-ordered proteins and large macromolecular assemblies breaking resolution barriers for studies of allosteric events and allosteric drug discovery [113,114,115]. The processing of increasingly large, complex datasets using traditional data processing strategies is exceedingly expensive in both user time and computational resources. 

The recent development of data processing capitalizes on AI and ML models to enhance the efficacy of data analysis and validation and automate the steps of particle picking, 3D map reconstruction, and local resolution determination [116]. Furthermore, DL algorithms have been used to improve the accuracy of cryo-EM image reconstruction, by providing more accurate estimates of the resolution and signal-to-noise ratio. DL-based image processing approaches have been used in processes of cryo-electron microscopy 3D reconstruction and atomic structure determination [117,118,119,120]. CryoDRGN (Deep Reconstructing Generative Networks) is a network that enables heterogeneous reconstruction by learning a deep generative model of 3D structure from diverse cryo-EM density maps. This neural network representation of structure can model single density maps with a higher resolution, and it can be used for unsupervised heterogeneous reconstruction [121]. Cryo-EM reconstructs multiple configurations of the same sample from images, which usually needs several rounds of 2D and 3D classifications to understand the conformational heterogeneity. CryoDRGN can produce a large number of 3D density maps, and automated processing methods for analyzing the results of CryoDRGN have also been created [122]. ResNet is a type of deep learning model that uses a deep neural network architecture composed of residual layers. It has been shown to be effective in a variety of computer vision and natural language processing tasks. The ResNet model uses a set of convolutional layers to learn the representation of the input data, and then a set of residual layers to refine the representation. The residual layers are used to learn the difference between the input and the output of the convolutional layers, making the model more efficient and accurate. The DeepHEMNMA method uses this architecture to learn the distribution of the conformational heterogeneity captured in the single particle images and to use this distribution to generate a single, ensemble-averaged structure [123].

Single-molecule Förster resonance energy transfer (smFRET) paved the way for studying dynamics in macromolecular structures under biologically relevant conditions [124] The recent breakthroughs in smFRET technologies have enabled dynamic studies of large biomolecules at atomic resolution. MD simulations have been widely applied with smFRET experiments to provide atomistic insights into the dynamic behavior of biomolecules [125,126]. 

Microsecond MD simulations have been used in combination with ML methods and restraints derived from smFRET experiments to successfully recapitulate and interpret smFRET states and allosteric conformational transitions [127,128,129]. This integration of single-molecule experiments, MD simulations, and ML models involves supervised learning, in which an initial Markov State Model (MSM) is constructed from raw simulation data, and unsupervised learning, which entails performing hidden Markov modeling to optimize the initial MSM using smFRET time-series measurements [127]. MD simulations can be used in conjunction with information from smFRET experiments to create an automated FRET-assisted structural model that can increase the accuracy of an integrated structure, as well as allow for FRET-assisted coarse-grained structural modeling and MD simulation-based refinement [130]. Recently, ML developments in single-molecule imaging automation and single-molecule feature recognition, including smFRET intensity trace analysis, have been reviewed [131].

The DeepFRET method utilizes DL architectures for rapid and automated classification of smFRET data, which is a significant challenge in smFRET experiments and the analysis of protein dynamics [132]. This approach, trained on simulated smFRET time-series, accurately and effectively distinguishes and categorizes smFRET experimental series both in simulated ground truth data and in a real-world dataset. The AutoSiM approach, which is based on DL, is used for automated screening of smFRET data to identify high-quality traces for further investigation [133].

By leveraging smFRET and MD simulations, researchers have explored the molecular basis and dynamic events associated with allosteric regulatory mechanisms. For example, a study of the NMDA receptor uncovered the mechanism of allostery underlying negative cooperativity, revealing that binding of one agonist leads to flexibility and increased conformational spread at the second agonist site [134]. Additionally, smFRET imaging tools have been used to analyze the conformational dynamics and allosteric modulation of the SARS-CoV-2 Spike protein by antibody binding [135].

By combining smFRET, fluorescence correlation spectroscopy (FCS), and fluorescence lifetime measurements with atomistic MD simulations, researchers were able to show how ATP hydrolysis in Hsp90 chaperones induces allosteric changes at a distant protein binding site and modulates chaperone activities [136]. Furthermore, the effects of positive allosteric modulators on structural dynamics of metabotropic glutamate receptors (mGlu2) were explored in optimized detergent micelles using smFRET at sub-millisecond timescales, demonstrating how the modulators can increase mGlu2 receptor efficacy through fast inter-subunit rearrangements [137].

Despite considerable progress in applications of AI and ML methods in cryo-EM and smFRET studies, the intersection between allosteric regulation, the emerging structural biology technologies, and AI approaches are largely unexplored, calling for the development of AI-augmented integrative structural biology. This is an exciting area of research, with the potential to reveal the underlying mechanisms of allosteric regulation and to develop new ways of controlling the behavior of proteins. By better understanding the mechanisms of allosteric regulation and developing new AI approaches, we can gain a better understanding of how proteins work and how they can be manipulated to produce desired biological outcomes. In addition, by combining these technologies, AI-enabled integrative tools could unveil the invisible aspects of protein ‘life’ revealing hidden protein states and reconstructing allosteric landscapes for therapeutically important regulatory proteins operating under allosteric control. Through potential synergies of these innovative technologies, AI-enhanced biochemical and structural biology tools can create novel ways of controlling the structure and activity of proteins to achieve desired outcomes, leading to a new era of protein engineering and synthetic biology.

## 7. Concluding Remarks and Future Perspectives

Despite the accepted understanding that intricate protein systems and regulatory complexes often function as dynamic and diverse allosteric machines, the characterization of hidden and uncommon protein functional states, allosteric structural changes, and allosteric pathways is still surprisingly scarce, necessitating the combination of new structural, biophysical, and computational approaches to address these problems. Research in the area of allosteric mechanisms is advancing in several new directions, such as the use of computational methods for the detection and visualization of allosteric networks, as well as the development of novel experimental methods to study allosteric processes. The latest remarkable progress in deep high-throughput mining and comprehensive mapping of allosteric protein landscapes and allosteric mechanisms using novel experimental technologies created a wealth of high-quality data that were used in the development of new experiment-guided AI systems. The recent studies have shown that the continuous developments and adaptations of AI and ML tools in high throughput biochemistry and their integration with DMS tools can reveal atomistic details of allosteric landscapes and result in a comprehensive characterization of allosteric mutations, yielding unprecedented insights into molecular mechanisms of complex biological systems.

The latest advances in structural characterization of allosteric molecular events and hidden functional states using cryo-EM, NMR, smFRET spectroscopy have highlighted the growing need for data-centric integrative biophysics approaches. These emerging methods and tools, including high-throughput sequencing, microscopy imaging, proteomics, metabolomics, and systems biology, involve utilization of AI and ML models trained on large datasets from different sources. AI-augmented integrative biophysics approaches leverage the power of AI to facilitate the integration of multiple data sources and to discover novel physical principles underlying biological systems. The latest developments reviewed in this study suggest that AI can be used to develop more accurate predictive models of diverse and complex biological systems, identify relevant features from large scale datasets, and provide insights into the underlying physical mechanisms of biological processes. AI-augmented biophysics approaches in structural biology can also be used to automate the experimental design process and optimize laboratory techniques, allowing scientists to conduct research and identify meaningful patterns in the data quickly and efficiently. In addition, AI-augmented integrative biophysics approaches will provide new strategies for drug design and development—from allosteric drug discovery to pathway-targeted design, systems medicine, and protein engineering applications. By developing an open science infrastructure for machine learning studies of allosteric regulation and validating computational approaches using integrative studies of allosteric mechanisms, the scientific community can expand the toolkit of approaches for dissecting and interrogation allosteric mechanisms in many therapeutically important proteins. The development of community-accessible tools that uniquely leverage the existing experimental and simulation knowledgebase to enable interrogation of the allosteric functions can provide a much-needed boost to further innovation and integration of experimental and computational technologies empowered by a booming AI field.

## Data Availability

Data are fully contained within the article. Crystal structures were obtained and downloaded from the Protein Data Bank (http://www.rcsb.org) (accessed on 5 April 2023). The rendering of protein structures was done with interactive visualization program UCSF ChimeraX package (https://www.rbvi.ucsf.edu/chimerax/) and Pymol (https://pymol.org/2/) (accessed on 5 April 2023). All the software tools are freely available in the GitHub sites https://github.com/smu-tao-group; https://github.com/smu-tao-group/protein-VAE; https://github.com/smu-tao-group/PASSer2.0; https://github.com/stevenagajanian/KInGAN; and https://github.com/kassabry/Perturbation_Experiment (accessed on 12 April 2023).
